# Manganese-coordinated mRNA vaccines with enhanced mRNA expression and immunogenicity induce robust immune responses against SARS-CoV-2 variants

**DOI:** 10.1126/sciadv.abq3500

**Published:** 2022-12-23

**Authors:** Na Fan, Kepan Chen, Rong Zhu, Zhongwei Zhang, Hai Huang, Shugang Qin, Qian Zheng, Zhongshan He, Xi He, Wen Xiao, Yupei Zhang, Yongjun Gu, Changchun Zhao, Yongmei Liu, Xin Jiang, Shuaicheng Li, Yuquan Wei, Xiangrong Song

**Affiliations:** ^1^ Department of Critical Care Medicine, Frontiers Science Center for Disease-related Molecular Network, State Key Laboratory of Biotherapy, West China Hospital, Sichuan University, Chengdu, Sichuan, China.; ^2^ WestChina-Frontier PharmaTech Co. Ltd., Chengdu, Sichuan, China.; ^3^ Department of Computer Science, City University of Hong Kong, Tat Chee Ave., Kowloon Tong, Hong Kong, China.

## Abstract

It is urgent to develop more effective mRNA vaccines against the emerging severe acute respiratory syndrome coronavirus 2 (SARS-CoV-2) variants owing to the immune escape. Here, we constructed a novel mRNA delivery system [IC8/Mn lipid nanoparticles (IC8/Mn LNPs)]with high immunogenicity, via introducing a stimulator of interferon genes (STING) agonist [manganese (Mn)] based on a newly synthesized ionizable lipid (IC8). It was found that Mn can not only promote maturation of antigen-presenting cells via activating STING pathway but also improve mRNA expression by facilitating lysosomal escape for the first time. Subsequently, IC8/Mn LNPs loaded with mRNA encoding the Spike protein of SARS-CoV-2 Delta or Omicron variant (IC8/Mn@D or IC8/Mn@O) were prepared. Both mRNA vaccines induced substantial specific immunoglobulin G responses against Delta or Omicron. IC8/Mn@D displayed strong pseudovirus neutralization ability, T helper 1–biased immune responses, and good safety. It can be concluded that IC8/Mn LNPs have great potential for developing Mn-coordinated mRNA vaccines with robust immunogenicity and good safety.

## Teaser

Manganese acts as adjuvant and mRNA translation enhancer to improve the immune responses of mRNA vaccines.

## INTRODUCTION

Coronavirus disease 2019 (COVID-19), caused by severe acute respiratory syndrome coronavirus 2 (SARS-CoV-2) and its variants, has led to a severe global public health crisis (*1*–*6*). Several vaccines have been approved to prevent against COVID-19, including two mRNA vaccines, COMIRNATY (BNT162b2 of Pfizer/BioNTech) and SPIKEVAX (mRNA-1273 of Moderna) *(**7*, *8**).* The mRNA vaccines have unique advantages such as high immunogenicity, rapid manufacturing, and low cost, proving extremely effective to fight COVID-19. However, the immune escape of the emerging variants of SARS-CoV-2 is challenging the present COVID-19 mRNA vaccines. Therefore, it is imperative to develop more effective mRNA vaccines against the new SARS-CoV-2 variants.

Efficient antigen presentation by antigen-presenting cells (APCs), such as dendritic cells (DCs), a prerequisite for the high efficacy of mRNA vaccines, requires sufficient protein translation and APCs maturation *(**9*–*11**).* Furthermore, highly expressed costimulatory molecules on mature DCs provide a secondary signal for the activation of naive T cells (*12*–*14*). Adjuvants can promote DCs maturation and up-regulate the costimulating molecule expression of DCs. Therefore, the adjuvant codelivery is an effective strategy to enhance the immune effects of mRNA vaccines.

The agonists of Toll-like receptors (TLRs) and stimulator of interferon genes (STING), as adjuvants, have been applied in mRNA vaccines. For example, Kübler *et al.* (*15*) reported a CV9103 prostate cancer vaccine containing self-adjuvanted mRNA (RNActive) with activation of TLR7, which induced broader immune responses and thereby prolonged the survival of patients with castration-resistant prostate cancer. TriMix (a TLR4 agonist, containing CD40L, CD70, and TLR4a mRNA) was used to construct the mRNA vaccine iHIVARNA-01 to prevent chronic HIV infection (*16*–*18*). Islam *et al.* (*19*) prepared the mRNA vaccine nanoparticle consisting of an ovalbumin-coded mRNA (OVA mRNA) and a palmitic acid–modified TLR7/8 agonist R848 (C16-R848), which increased the effectiveness of mRNA vaccines for cancer immunotherapy. However, TLR agonists can cause severe cytokine storms, which limit their clinical application (*20*–*22*). Recently, STING agonists have been verified to induce relatively low levels of local and systemic inflammation when used as adjuvants (*23*–*25*). Miao *et al.* (*25*) developed a combinatorial library of ionizable lipid-like materials as mRNA delivery vehicles that facilitated mRNA delivery in vivo and enhanced antitumor efficacy via activating the STING pathway. Manganese (Mn), an essential element in many physiological processes, was reported to stimulate the STING pathway by directly activating cyclic guanosine 5′-monophosphate (GMP)–adenosine 5′-monophosphate (AMP) synthase (cGAS) to synthesize 2′ 3′-cyclic GMP-AMP (2′ 3′-cGAMP) (*26*, *27*). However, it remains unclear whether Mn can enhance the immune effect of mRNA vaccines.

The delivery systems protecting mRNA from degradation and allowing cellular uptake and mRNA release are indispensable for efficient mRNA expression in vivo (*11*, *28*, *29*). Lipid nanoparticles (LNPs) have shown good efficacy and safety in the successful clinic application of BNT162b2 and mRNA-1273 (*30*, *31*). We previously found that the ionizable lipid in LNPs notably influenced the mRNA expression. The ionizable lipids with multiple tertiary amino nitrogen atoms, compared to the ones with one nitrogen atom like mRNA-1273, could remarkably enhance the immunogenicity of mRNA vaccines (*32*).

**Fig. 1.**
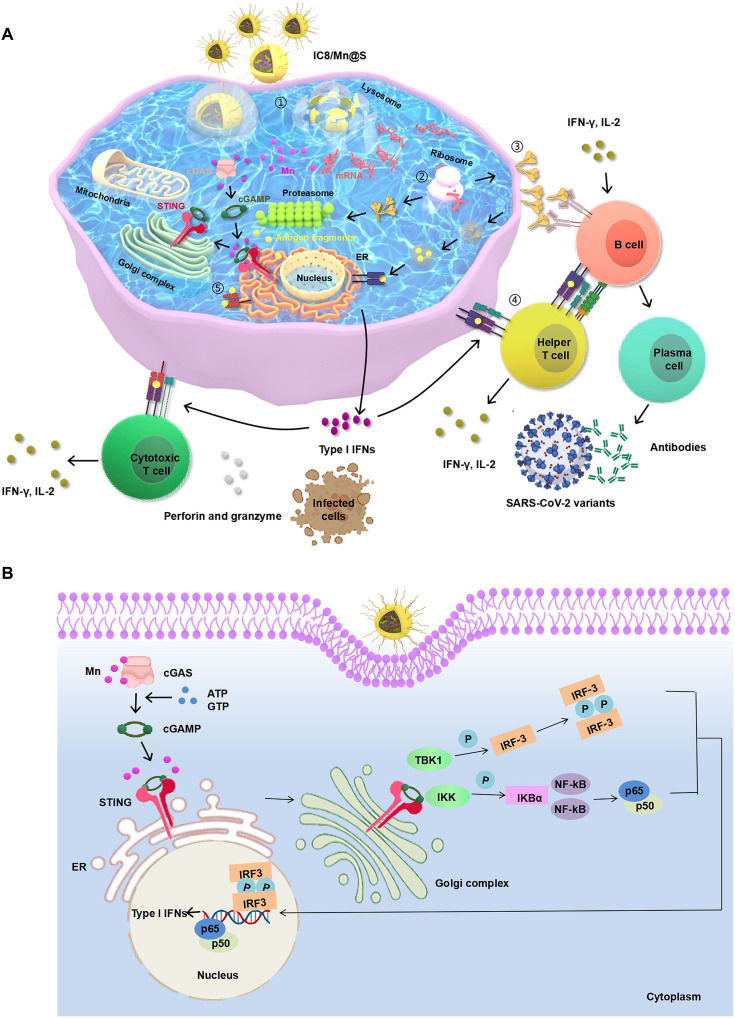
Schematic illustration of IC8/Mn@S against the SARS-CoV-2 variants and the activation of the STING pathway. (**A**) ① IC8/Mn@S is endocytosed by APCs. ② After escaping from lysosome, S mRNA and Mn are released in the cytosol, mRNA is translated into protein by the ribosomes, and Mn activates the STING pathway. The translated antigenic protein can activate immune responses in several ways. ③ After obtaining the first signal from S protein via B cell antigen receptor and the second signal from activated helper T cells via CD40, B cells are activated and differentiate into plasma cells, which produce Spike-specific antibodies to neutralize SARS-CoV-2 variants. ④ The secreted antigens can be endocytosed by APCs again, degraded in endosomes, and presented to helper T cells by major histocompatibility complex class II (MHC II) proteins. Helper T cells stimulate B cells to produce neutralizing antibodies. ⑤ Intracellular antigens are broken down into smaller fragments by the proteasome, and the fragments are presented to cytotoxic T cells by major histocompatibility complex class I (MHC I) proteins. Activated cytotoxic T cells kill infected cells by secreting perforin and granzyme. ER, endoplasmic reticulum. (**B**) Mn activates the STING pathway. Mn directly activates cGAS, independent of double-stranded DNA, to synthesize 2′3′-cGAMP in the presence of adenosine 5′-triphosphate (ATP) and guanosine 5′-triphosphate (GTP). Then, 2′3′-cGAMP activates protein STING, and Mn can enhance the binding ability of 2′3′-cGAMP and STING. Subsequently, STING translocates from ER to Golgi complex, where STING activates TANK binding kinase 1 (TBK1), which phosphorylates interferon regulatory factor 3 (IRF-3). Last, phosphorylate IRF-3 enters nucleus and initiates the transcription of type I IFNs, which can stimulate the maturation of APCs, promote the differentiation of helper T cells, and activate cytotoxic T cells. IKK, IκB kinase; NF-κB, nuclear factor kappa-light-chain-enhancer of activated B cells; IKBα, NF-kappa-B inhibitor alpha; P, phosphorylate.

Here, we designed and constructed novel LNPs (IC8/Mn LNPs) containing Mn and a newly synthesized ionizable lipid (IC8). The delivery of mRNA by IC8/Mn LNPs was first investigated, and then the immunogenicity of IC8/Mn LNPs loaded with mRNA (IC8/Mn@S) encoding the full-length Spike protein of SARS-CoV-2 variants was assessed. The mechanism protecting against the SARS-CoV-2 variants was also demonstrated (Fig. 1). Mn was found to improve the mRNA expression by facilitating lysosomal escape and stimulate APCs maturation by activating the STING pathway. Moreover, IC8/Mn@S with the optimal dosage of Mn (Mn/mRNA 1:1, w/w) induced robust and durable immunoglobulin G (IgG) antibodies against SARS-CoV-2 variants (Delta and Omicron). Hence, we constructed a novel highly efficient mRNA delivery system (IC8/Mn LNPs) with enhanced mRNA expression and immune response, which could have promising clinical applications in mRNA vaccines.

## RESULTS

### Preparation and characterization of IC8@mRNA and IC8/Mn@mRNA

**Fig. 2.**
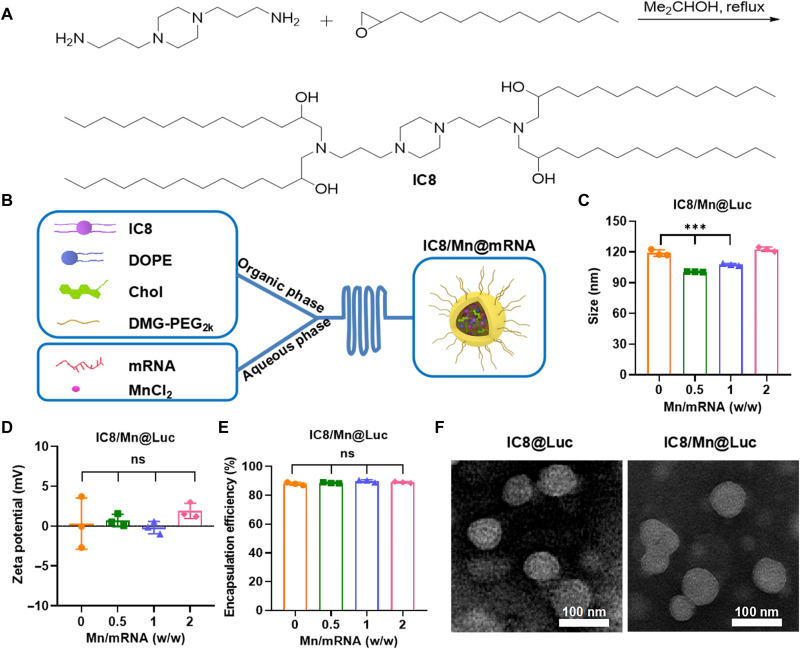
Preparation and characterization of IC8/Mn@mRNA. (**A**) The synthesis of IC8. (**B**) The schematic diagram of IC8/Mn@mRNA preparation by a microfluidic device. DOPE, 1,2-distearoyl-snglycero-3-phosphoethanolamine; DMG-PEG_2k_, 1,2-dimyristoyl-rac-glycero-3-methoxypolyethyleneglycol-2000. (**C** to **E**) The average size, zeta potential, and encapsulation efficiency of IC8/Mn@Luc containing different mass ratios of Mn to mRNA. (**F**) Representative TEM images of IC8@Luc and IC8/Mn@Luc. Scale bars, 100 nm. Statistical significance was tested using a one-way analysis of variance (ANOVA) among groups. ***P < 0.001; ns, not significant.

The efficient antigen presentation of APCs is indispensable for mRNA vaccines, which requires adequate protein expression and APCs maturation (*11*, *33*). Adjuvants are vital to improve vaccine immune responses because they can promote APCs maturation (*34*, *35*). However, excessive adjuvant-produced type I interferons (I-IFNs) may suppress mRNA translation and expression (*12*, *36*). Therefore, it is critical to optimize the dosage of adjuvants in mRNA vaccines. Here, the STING agonist Mn was introduced in LNPs based on a newly synthesized ionizable lipid (IC8) to construct a novel mRNA delivery system, IC8/Mn LNPs (Fig. 2A and fig. S1). The formulation of LNPs containing IC8 had been optimized in our previous study, so as to show good transfection in mRNA delivery (*32*). Therefore, we prepared IC8 LNPs loading luciferase mRNA (IC8@Luc) and IC8/Mn LNPs loading Luc mRNA (IC8/Mn@Luc) (containing a different mass ratio of Mn to mRNA) using a microfluidic device (Fig. 2B). Table S1 displayed the formulations of IC8@Luc and IC8/Mn@Luc. After ultrafiltration, in IC8/Mn@Luc, the mass ratio of Mn to mRNA changed from 0, 2.5, 5, and 10 to 0, 0.5, 1, and 2, respectively. The encapsulation efficiency of Mn in IC8/Mn@Luc was approximately 20%. We speculated that Mn was trapped in IC8/Mn@Luc because of its coordination interaction with the mRNA. As we can see in Fig. 2 (C to E), although the particle size of IC8/Mn@Luc (Mn/mRNA 0.5),w/w] containing a small amount of Mn decreased compared to IC8@Luc, the particle size of IC8/Mn@Luc increased with the increase of Mn content. The addition of different Mn contents affected insignificantly zeta potential (~0 mV) and mRNA encapsulation efficiency (~90%) of IC8/Mn@Luc compared to IC8@Luc (Fig. 2, C to E). The transmission electron microscopy (TEM) revealed that IC8@Luc and IC8/Mn@Luc were spherical with a dense core of diameter approximately 80 nm (Fig. 2F).

### In vitro and in vivo delivery of IC8@mRNA and IC8/Mn@mRNA

**Fig. 3.**
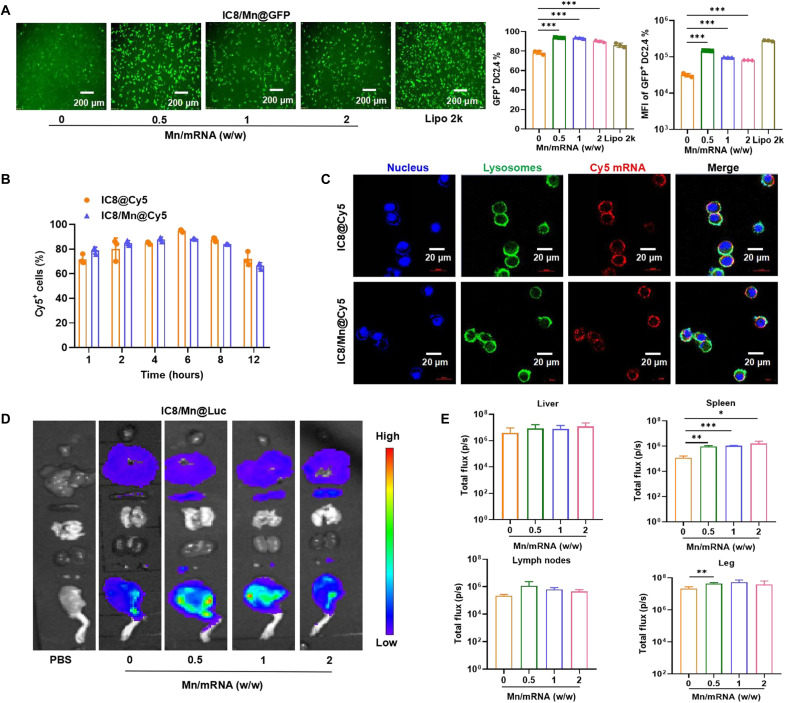
In vitro and in vivo delivery of IC8/Mn@mRNA. (**A**) GFP mRNA was transfected into DC2.4 cells using IC8/Mn LNPs or Lipofectamine 2000 (Lipo 2k). Cells were harvested after 24 hours of incubation. GFP expression was visualized using inverted fluorescence microscopy and quantified by flow cytometry. (**B**) Cellular uptake of IC8@Cy5 and IC8/Mn@Cy5 at different times was quantified by flow cytometry. (**C**) Confocal fluorescence images of DC2.4 cells incubated with IC8@Cy5 and IC8/Mn@Cy5 in 10% fetal bovine serum (FBS) medium for 5 hours. DAPI (4′,6-diamidino-2-phenylindole; blue) was used to track the cell nuclei, and LysoTracker Green DND-26 (green) was used to track lysosomes; red fluorescence was from Cy5 mRNA. Colocalization analysis of the different channels in “Merge.” (**D** and **E**) Comparison of biodistribution between IC8 LNPs and IC8/Mn LNPs encapsulated with 20 μg of Luc mRNA after intramuscular administration for 6 hours. BALB/c mice were euthanized, and biodistribution and bioluminescence intensity in major organs were detected with an IVIS Lumina system. The data are shown as means ± SD. Statistical significance was tested using ANOVA among groups. *P < 0.05, **P < 0.01, and ***P < 0.001.

The mRNA encoding green fluorescent protein (GFP) was applied as a reporter gene to assess whether introducing Mn affected the expression of mRNA vaccines in DC2.4 cells and bone marrow–derived DCs (BMDCs). IC8/Mn@Luc was served as a scrambled mRNA control, and no fluorescence was observed. The fluorescence intensity of IC8/Mn@GFP containing different Mn contents all increased compared to IC8@GFP in DC2.4 cells, which were visually shown by inverted fluorescence microscopy (Fig. 3A). However, the fluorescence intensity decreased with increasing Mn content in IC8/Mn@GFP. In addition, we adopted flow cytometry to quantify the transfection efficiency and mean fluorescence intensity (MFI) of IC8@GFP and IC8/Mn@GFP. Data showed that the transfection efficiency and MFI of IC8/Mn@GFP containing different Mn contents were higher than those of IC8@GFP. The transfection efficiency and MFI of IC8/Mn@GFP showed a downward trend with increasing Mn content in IC8/Mn@GFP (Fig. 3A). Furthermore, BMDCs with high purity (~80%) and low maturity (~10%) were extracted for mRNA transfection (fig. S2A). Although the BMDCs rarely transfect, 20 to 30% transfection efficiency of IC8/Mn@GFP was detected, while no transfection of IC8@GFP was observed. The transfection efficiency and MFI of IC8/Mn@GFP containing different Mn contents in BMDCs showed the same trend as DC2.4 cells (fig. S2, B and C). The experiments suggested that adding Mn improved the transfection efficiency of the mRNA; however, the increase in the Mn content decreased the transfection efficiency of the mRNA. We speculated that the phenomenon was due to the fact that increasing the Mn content in IC8/Mn@GFP enhanced the ability of IC8/Mn@GFP to activate the STING pathway, thus producing more IFN-β. It has been reported that IFN-β might inhibit mRNA translation (*12*, *36*). Therefore, it was necessary to screen the optimal Mn content in mRNA vaccines.

We conducted several mechanistic studies to explore how Mn could improve the transfection efficiency and MFI of IC8/Mn LNPs. First, we investigated whether cellular internalization was associated with transfection efficiency, which can be one major obstacle in the transfection process for mRNA vaccines (*37*, *38*). We studied cellular uptake in DC2.4 cells by comparing the IC8 LNPs and IC8/Mn LNPs loaded with Cyanine 5 (Cy5)–labeled mRNA, denoting them as IC8@Cy5 and IC8/Mn@Cy5, respectively. The flow cytometry data indicated that IC8@Cy5 and IC8/Mn@Cy5 had similar cellular uptake from 1 to 12 hours, and the percentage of cells positive for Cy5 mRNA approached 100% at 6 hours (Fig. 3B). We speculated that the cellular uptake of IC8@Cy5 and IC8/Mn@Cy5 containing 0.5 μg of Cy5 mRNA may reach saturation in cells, so the proportion of Cy5-positive cells decreased because of cell proliferation at 12 hours. Furthermore, three cellular uptake inhibitors were adopted—the chlorpromazine (CPZ) clathrin–mediated uptake inhibitor, caveolin-mediated uptake inhibitor nystatin (NS), and the macropinocytosis uptake inhibitor cytochalasin D (CD)—to elucidate the endocytic mechanism of IC8@Cy5 and IC8/Mn@Cy5 in DC2.4 cells. As shown in fig. S2D, IC8@Cy5 and IC8/Mn@Cy5 also behaved similarly to these inhibitors. CPZ and NS impeded cellular uptake to the largest extent, indicating that IC8@Cy5 and IC8/Mn@Cy5 were predominantly internalized via clathrin-mediated and caveolin-mediated pathways. Hence, IC8@Cy5 and IC8/Mn@Cy5 had similar cellular uptake properties, suggesting that other factors were responsible for their different transfection efficiencies.

Second, we examined the lysosomal escape abilities of IC8@Cy5 and IC8/Mn@Cy5. When mRNA vaccines enter cells through the clathrin-mediated or macropinocytosis cellular uptake pathway, mRNA vaccines are transported to lysosomes (*39*). If mRNA vaccines cannot escape from the lysosomes in time, then they will break down in lysosomes, resulting in low transfection efficiency (*40*, *41*). Therefore, effective mRNA vaccines should have good ability of lysosomal escape. Mn can promote the lysosomal escape of nanoparticles by enhancing pH buffering capacity (*42*). We observed the lysosomal escape of IC8@Cy5 and IC8/Mn@Cy5 through confocal microscopy after 5 hours of transfection. IC8/Mn@Luc was served as a scrambled mRNA control to see the background fluorescence, and there were no red signals in cells treated with IC8/Mn@Luc (fig. S2E). Meanwhile, there were no red signals in cells at 0 hours, indicating that IC8/Mn@Cy5 was not endocytosed into cells (fig. S2F). After 5 hours of transfection, IC8@Cy5 and IC8/Mn@Cy5 were endocytosed into cells. The intracellular distribution of Cy5 mRNA (red) and lysosomes (green) is shown in Fig. 3C. The red signals overlapped with the green signals to produce yellow signals, indicating that the mRNA vaccines were trapped in the lysosomes. Compared to IC8@Cy5, IC8/Mn@Cy5 showed clear red and green signal separations, confirming that IC8/Mn@Cy5 had better lysosomal escape ability. The Pearson’s correlation coefficient was used to further evaluate the lysosomal escape ability of IC8@Cy5 and IC8/Mn@Cy5 (*43*). The Pearson’s correlation coefficient of IC8@Cy5 was calculated to be about 0.45, demonstrating the colocalization of Cy5 mRNA and lysosomes, while the Pearson’s correlation coefficient of IC8/Mn@Cy5 was reduced to 0.1, showing that the IC8/Mn@Cy5 could efficiently escape from the lysosomes compared to IC8@Cy5 (fig. S2G). Therefore, Mn could improve the transfection efficiency of IC8/Mn@Cy5 by promoting lysosomal escape rather than improving cellular uptake.

Next, IC8@Luc and IC8/Mn@Luc were intramuscularly injected into BALB/c mice to explore the mRNA distribution and expression in vivo. Phosphate-buffered saline (PBS) served as a negative control. Representative images in bioluminescence and statistical data are shown in Fig. 3 (D and E), respectively. Compared to IC8@Luc, IC8/Mn@Luc significantly improved the intensity of bioluminescence in the spleen. At the injection site of the muscle, the bioluminescence intensity of IC8/Mn@Luc (Mn/mRNA 0.5, w/w) was the highest and IC8/Mn@Luc (Mn/mRNA 2, w/w) was the lowest, which showed a consistent mRNA expression trend in vitro (Fig. 3A). In summary, these data indicated that IC8/Mn LNPs performed favorably for mRNA delivery in vitro and in vivo and were applicable for mRNA vaccines to prevent against SARS-CoV-2 variants.

### IC8/Mn@S induced strong and durable humoral responses with superior neutralizing activities against the SARS-CoV-2 variants

**Fig. 4.**
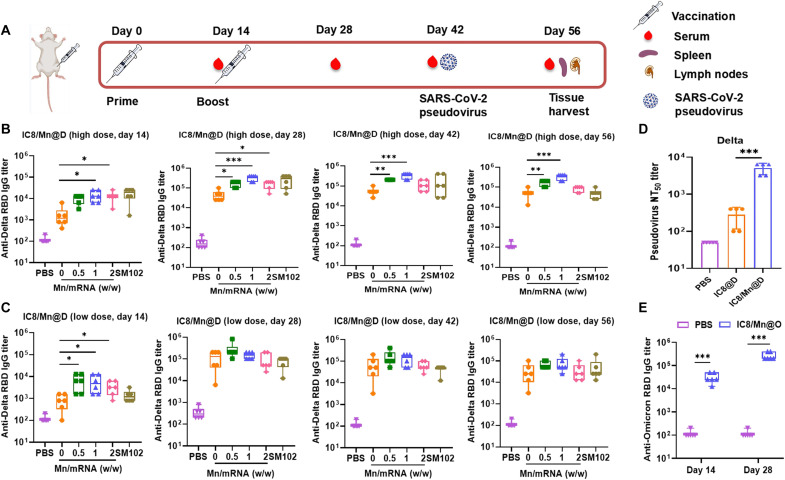
Mouse immunization schedule and humoral immune response induced by IC8/Mn@D containing different mass ratios of Mn to mRNA. (**A**) Schematic diagram of immunization and sample collection. BALB/c mice were immunized twice with IC8/Mn@D containing 10 or 30 μg of Delta mRNA on day 0 and day 14. Mice in the PBS group were administrated with PBS as control. Serum samples from IC8/Mn@D (containing 10 or 30 μg of Delta mRNA)–immunized mice were collected every 2 weeks, and anti-Delta receptor binding domain (RBD) IgG titers were measured on day 14, day 28, day 42, and day 56 by ELISA. (**B** and **C**) The anti-Delta RBD IgG titers of IC8/Mn@D-immunized mice were determined by ELISA. (**D**) The serum samples collected from IC8/Mn@D (Mn/mRNA 1:1, w/w, containing 30 μg of Delta mRNA)–immunized mice at day 42 were used to test neutralizing ability against SARS-CoV-2 Delta pseudovirus. (**E**) BALB/c mice were immunized twice with IC8/Mn@O (Mn/mRNA 1:1, w/w) containing 30 μg of Omicron mRNA at day 0 and day 14. Serum samples from IC8/Mn@O-immunized mice were collected at day 28, and the anti-Omicron RBD IgG was measured by ELISA. The data are shown as means ± SD. Statistical significance was tested using ANOVA among groups. *P < 0.05, **P < 0.01, and ***P < 0.001.

We loaded the mRNA encoding the full-length Spike protein of the SARS-CoV-2 Delta variant (Delta mRNA) into IC8 LNPs and IC8/Mn LNPs, referred to as IC8@D and IC8/Mn@D, respectively, to check efficiency. The S protein expressions of IC8@D and IC8/Mn@D were evaluated by Western blot (WB) after transfection of human embryonic kidney (HEK) 293T cells. The WB results indicated that IC8/Mn@D expressed more S protein than IC8@D, which aligned with the results of the in vitro transfection (fig. S3). The immunogenicity and protective potentials of IC8@D, IC8/Mn@D, and SM102@D were assessed in immunized BALB/c mice. SM-102 was the ionizable lipid used in mRNA-1273. SM102@D, as the positive control, was the Delta mRNA–loaded LNPs with the same formulation of mRNA-1273. Groups of mice (*n* = 6) were inoculated with IC8@D, IC8/Mn@D, and SM102@D intramuscularly twice on day 0 and day 14 with a high dose (30 μg of Delta mRNA) or low dose (10 μg of Delta mRNA). The serum samples of IC8@D-, IC8/Mn@D-, and SM102@D-immunized mice were collected at different time points and analyzed by enzyme-linked immunosorbent assay (ELISA) (Fig. 4A). The IgG titers of all mRNA vaccines, including IC8@D, IC8/Mn@D, and SM102@D, increased from day 14 to day 28 and peaked at day 28. The IgG titers of IC8/Mn@D with different Mn contents all increased compared to IC8@D (Fig. 4, B and C). However, the trends of variation in IgG titers of IC8/Mn@D with high and low doses differed with the increase of Mn content. At the low dose, IgG titers decreased with the increasing Mn content in IC8/Mn@D, and IC8/Mn@D (Mn/mRNA 0.5:1, w/w) induced the best IgG titer. However, IC8/Mn@D (Mn/mRNA 1:1, w/w) had the best IgG titer at the high dose. The IgG titers of IC8/Mn@D (Mn/mRNA 2,w/w) and SM102@D reduced from 28 to 56 days. In contrast, the IgG titer of IC8/Mn@D (Mn/mRNA 1:1, w/w) remained roughly constant from 28 to 56 days, indicating that IC8/Mn@D (Mn/mRNA 1:1, w/w) could maintain high and durable immune responses. In conclusion, at the low dose, Mn had a minor effect as an adjuvant due to the low content of Mn in IC8/Mn@D. The IgG titer levels of IC8/Mn@D containing different Mn contents were concordant with the mRNA expression levels. However, at the high dose, the Mn contents in IC8/Mn@D were sufficient to exert the adjuvant effect, so the IgG titer levels of IC8/Mn@D containing different Mn contents were not only related to mRNA expression but also related to the adjuvant effect.

Furthermore, we adopted a pseudovirus neutralization assay to assess whether sera collected on day 42 from mice twice vaccinated were able to neutralize the pseudovirus of the SARS-CoV-2 Delta variant. The NT_50_ titer (50% neutralization titers) of optimal IC8/Mn@D (Mn/mRNA 1:1, w/w) was 6400, significantly higher than the NT_50_ titer of IC8@D (400). These results indicated that IC8/Mn@D-vaccinated mice could induce enhanced neutralizing antibodies against the pseudovirus of the SARS-CoV-2 Delta variant (Fig. 4D).

In addition, we prepared mRNA vaccine IC8/Mn@O (Mn/mRNA 1:1, w/w) by loading mRNA encoding the S protein of SARS-CoV-2 Omicron variant (Omicron mRNA) into IC8/Mn LNPs. As seen in Fig. 4E, the IgG titer of IC8/Mn@O increased from day 14 to day 28 and peaked at day 28. The IgG titer level of IC8/Mn@O was comparable to that of IC8/Mn@D, indicating that IC8/Mn LNPs were a universal mRNA delivery system.

### IC8/Mn LNPs promoted maturation and antigen presentation of DCs

**Fig. 5.**
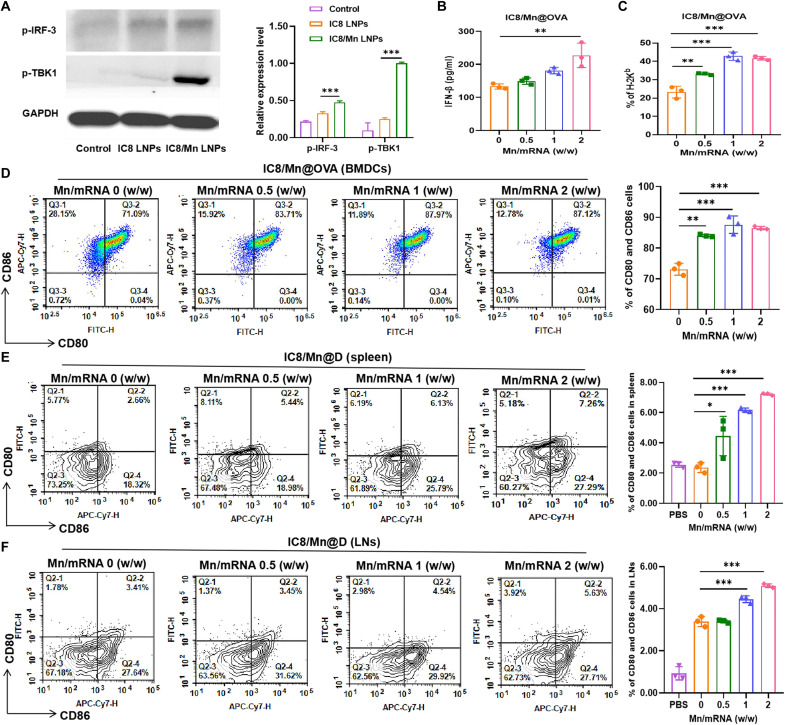
Effect of IC8/Mn LNPs on maturation and antigen presentation of DCs. (**A**) WB analysis of the STING pathway downstream proteins expressions (p-IRF-3 and p-TBK1) in DC 2.4 cells after treatment with PBS, IC8 LNPs, and IC8/Mn LNPs for 24 hours. GAPDH, glyceraldehyde-3-phosphate dehydrogenase. (**B**) ELISA examination of IFN-β secretion in the supernatant of BMDCs after treatment with IC8@OVA and IC8/Mn@OVA for 24 hours. (**C**) Flow cytometry analysis of SIINFEKL-H2K^b^ presentation on BMDCs with IC8@OVA and IC8/Mn@OVA for 24 hours. (**D**) Flow cytometry examination of BMDCs maturation (CD11c^+^CD80^+^CD86^+^) 24 hours after incubation with IC8@OVA and IC8/Mn@OVA. FITC, fluorescein isothiocyanate. (**E** and **F**) Flow cytometry examination of DCs maturation (CD11c^+^CD80^+^CD86^+^) in the spleen and lymph nodes 36 hours after intramuscular injection with IC8@D and IC8/Mn@D in BALB/c mice. The data are shown as means ± SD. Statistical significance was tested using ANOVA among groups. *P < 0.05, **P < 0.01, and ***P < 0.001.

As aforementioned, Mn can activate the STING pathway in two aspects as a safe STING agonist. When the STING pathway is activated, I-IFNs (especially IFN-β, the downstream signal of the STING pathway) are generated to promote DCs maturation and antigen presentation, thus enhancing cellular and humoral immune response and achieving better protection against the SARS-CoV-2 variants. We first verified that Mn in IC8/Mn LNPs could activate the cGAS-STING pathway by WB and ELISA. Its downstream markers, phosphorylate-TANK-binding kinase 1 (p-TBK1) and phosphorylate-interferon regulatory factor 3 (p-IRF-3), were used to verify the activation of the STING pathway of IC8/Mn LNPs. The results of WB and ELISA both showed that IC8/Mn LNPs could activate the STING pathway owing to the p-TBK1 and p-IRF-3 expression compared to control and IC8 LNPs (Fig. 5A and figs. S4 and S5). IFN-β in the supernatant of BMDCs increased with the increasing Mn content in IC8/Mn LNPs, further demonstrating that the activation degree of STING pathway was proportional to the Mn content in IC8/Mn LNPs (Fig. 5B).

Then, OVA mRNA(mRNA encoding model antigen) was used to evaluate the maturation and antigen presentation of BMDCs treated with IC8/Mn@OVA containing different contents of Mn in vitro. The maturation degree of BMDCs treated with IC8/Mn@OVA increased with the increasing Mn content in IC8/Mn@OVA (Fig. 5D and fig. S6). Similarly, the maturation degree of DCs isolated from the spleen and lymph nodes of IC8/Mn@D-vaccinated mice displayed enhanced maturation with increasing Mn contents in IC8/Mn@D (Fig. 5, E and F). The antigen presentation efficacy of the BMDCs was evaluated by detecting the frequency of the OVA (SIINFEKL)-H2K^b^ complex on the surface of the BMDC membranes. Upon incubation with the IC8/Mn@OVA, the fraction of the OVA-H2K^b^–positive BMDCs increased compared with IC8@OVA; however, the fraction of the OVA-H2K^b^–positive BMDCs did not increase with the increasing Mn content in IC8/Mn@OVA. IC8/Mn@OVA (Mn/mRNA 1:1, w/w) treatment induced optimal antigen presentation efficiency due to LNP-promoted cytosol release of OVA mRNA for antigen processing and presentation with the OVA/major histocompatibility complex class I (MHC I) complex (Fig. 5C). Combined with the results of in vitro transfection, when IC8/Mn LNPs contained a small amount of Mn (Mn/mRNA 0.5, w/w), Mn could maximize the efficiency of mRNA transfection but was inadequate to promote the maturation of DCs. When IC8/Mn LNPs contained a large amount of Mn (Mn/mRNA 2, w/w), more IFN-β was secreted via activation of the STING pathway, which could promote maturation of DCs but decrease mRNA transfection to some extent. Therefore, the optimal Mn usage dose in IC8/Mn LNPs (Mn/mRNA 1:1, w/w) struck a balance between mRNA expression and maturation of DCs, thereby achieving optimal antigen presentation. The results also explained the IgG titers of IC8/Mn@D with the high dose (Fig. 4B).

### IC8/Mn@D elicited strong T helper 1–biased T cell responses

**Fig. 6.**
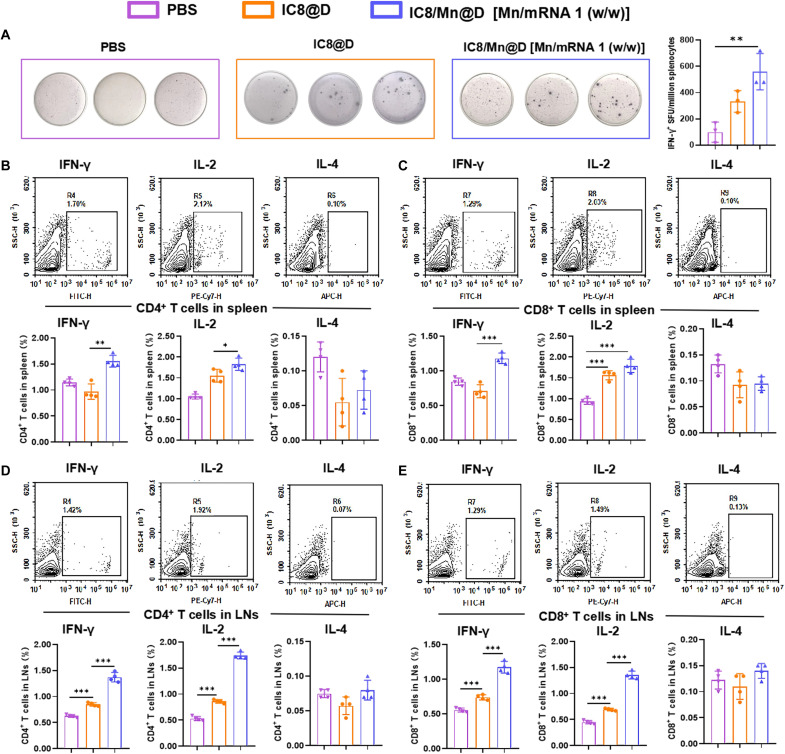
Induction of T_H_1-biased T cell response by IC8/Mn@D. (**A**) Splenocytes isolated from IC8@D-immunized mice and IC8/Mn@D-immunized mice on day 56 were stimulated with SARS-CoV-2 Delta S protein peptide pools for 36 hours, and IFN-γ–producing T cells were quantified with an ELISpot assay. (**B** to **E**) Splenocytes and lymph node cells (LNs) isolated from IC8@D-immunized mice and IC8/Mn@D-immunized mice at day 56 were stimulated with SARS-CoV-2 Delta S protein peptide pools for 12 hours, and Spike-specific cytokine-producing CD4^+^ T cells and CD8^+^ T cells were measured with an ICS. The data are shown as means ± SD. Statistical significance was tested using ANOVA among groups. *P < 0.05, **P < 0.01, and ***P < 0.001. SFU, spot forming units; SSC, side scatter; PE, phycoerythrin.

To evaluate cellular immune responses induced by IC8@D and IC8/Mn@D in mice, we carried out an enzyme-linked immunospot (ELISpot) assay and an intracellular cytokine staining (ICS) assay. Spleens of IC8@D-vaccinated mice and IC8/Mn@D-vaccinated mice were harvested on day 56. Splenocytes of IC8@D-vaccinated mice and IC8/Mn@D-vaccinated mice were isolated and restimulated with S peptide pools of the Delta variant of SARS-CoV-2 in vitro to evaluate the capacity to secrete IFN-γ by ELISpot (Fig. 6A). The results demonstrated that IFN-γ–secreting T cells from IC8@D- and IC8/Mn@D-immunized mice were more abundant than those from placebo-immunized mice. Likewise, IFN-γ–secreting T cells from IC8/Mn@D-immunized mice were more than those from IC8@D-immunized mice owing to the function of Mn.

Furthermore, splenocytes and lymph node cells from IC8@D-vaccinated mice and IC8/Mn@D-vaccinated mice were restimulated with S peptide pools from the Delta variant of SARS-CoV-2 in vitro to detect intracellular cytokine secretion [T helper 1 (T_H_1)–type cytokines: IFN-γ and interleukin-2 (IL-2); T_H_2-type cytokine: IL-4] of CD4^+^ T cells and CD8^+^ T cells. Compared with the placebo-immunized group, IC8@D and IC8/Mn@D induced more IFN-γ and IL-2 secretion by CD4^+^ T cells in splenocytes and lymph node cells rather than IL-4, which confirmed that IC8@D and IC8/Mn@D induced a T_H_1-biased immune response (Fig. 6, B to E, and fig. S7). Meanwhile, IC8/Mn@D induced more IFN-γ and IL-2 secretion by CD4^+^ T cells and CD8^+^ T cells in splenocytes and lymph node cells compared with IC8@D.

### Safety and stability profiles of IC8/Mn@D

**Fig. 7.**
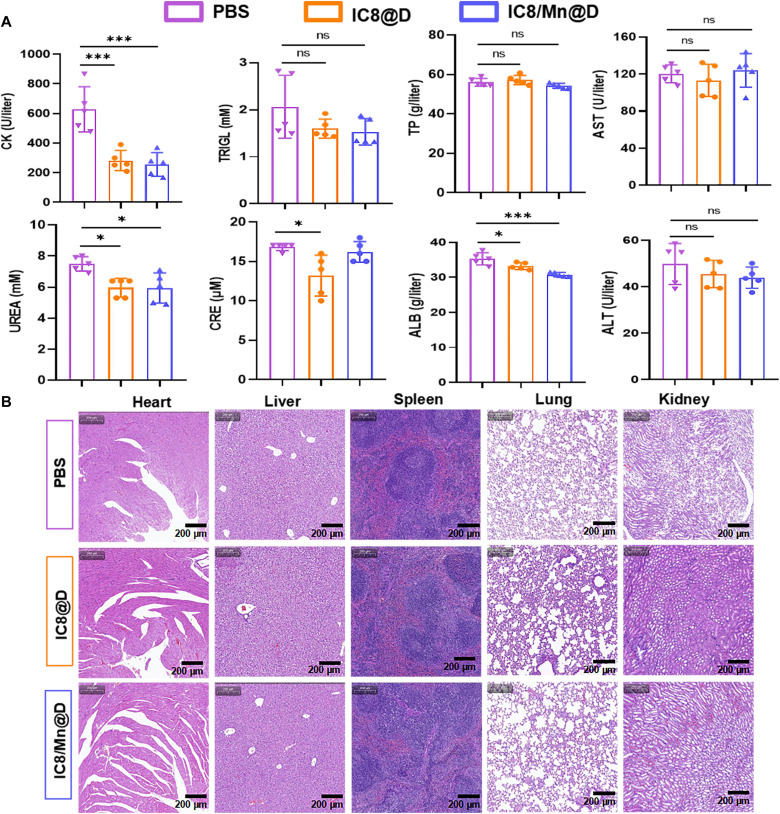
Safety of IC8/Mn@D. BALB/c mice were injected intramuscularly with IC8@D and IC8/Mn@D containing 30 μg of Delta mRNA for 24 hours to evaluate the safety of IC8@D and IC8/Mn@D. (**A**) Biochemical indicators of IC8@D- and IC8/Mn@D-vaccinated mice were detected by an automatic hematological biochemical analyzer. (**B**) H&E analyses of major organs from IC8@D- and IC8/Mn@D-vaccinated mice at day 56. Scale bars, 200 μm.

Although efficacy is critical for the development of vaccines, safety is undoubtedly more important. Therefore, an acute toxicity assay was applied to evaluate the preliminary safety of IC8@D and IC8/Mn@D (Mn/mRNA 1:1, w/w). IC8@D and IC8/Mn@D (Mn/mRNA 1:1, w/w) containing 30 μg of Delta mRNA were injected intramuscularly into BALB/c mice. Twenty-four hours later, serum samples were collected for biochemical analysis. No significant changes in biochemical indicators [triacylglycerol (TRIGL), total protein (TP), aspartic acid aminotransferase (AST), and alanine aminotransferase (ALT)] were observed in IC8@D- and IC8/Mn@D-vaccinated mice compared to mice in the PBS group (Fig. 7A). Other biochemical indicators [creatine phosphokinase (CK), urinary anhydride (UREA), creatinine (CRE), and albumin (ALB)] of IC8@D and IC8/Mn@D groups were lower than the PBS group, which had no clinical significance. The organs of each mouse (heart, liver, spleen, lung, and kidney) were harvested from IC8@D- and IC8/Mn@D-immunized mice at day 56 in the previous experiment for hematoxylin and eosin (H&E) staining. The images showed no noticeable histopathological differences in the main organs between the groups treated with IC8@D or IC8/Mn@D and the PBS group (Fig. 7B). These data confirmed that IC8/Mn@D had good safety.

Meanwhile, good stability is beneficial to the storage and transportation of mRNA vaccines. To study the stability of the vaccine, IC8/Mn@D (Mn/mRNA 1:1, w/w) had been stored at 4°C for 0, 7, 14, and 28 days and were observed for the changes of the particle size, zeta potential, mRNA encapsulation efficiency, and IgG titer. Mice (*n* = 6) were injected IC8/Mn@D (Mn/mRNA 1:1, w/w) intramuscularly twice with the high dose (30 μg of Delta mRNA). The serum samples were collected at day 28 after first administration and analyzed by ELISA. The results showed that the particle size, zeta potential, mRNA encapsulation efficiency, and IgG titer did not decrease significantly after storage at 4°C for 0, 7, 14, and 28 days, which demonstrated that IC8/Mn@D (Mn/mRNA 1:1, w/w) had superior stability (fig. S8).

## DISCUSSION

mRNA vaccines are rapidly developed and approved for the market during COVID-19 because of high effectiveness, good safety, and rapid production (*13*, *44*, *45*). However, with the emergence of new SARS-CoV-2 variants, the approved mRNA vaccines may not provide comprehensive protection owing to the immune escape (*8*, *46*). It is necessary to develop more effective mRNA vaccines against SARS-CoV-2 variants.

Naked mRNA is a bioactive macromolecule with a negative charge, which is extremely unstable in the physiological environment. Meanwhile, naked mRNA cannot penetrate cell membranes and target cells or tissues (*45*). Effective and safe delivery systems can protect mRNA from being degraded and improve mRNA expression via promoting mRNA cellular uptake or lysosomal escape and enhance antigen presentation of APCs, which are essential for mRNA vaccines to provoke immune responses. At present, cationic lipids, polymers, and nucleoside lipids are applied for compressing and encapsulating mRNA (*22*, *30*, *40*). In particular, LNPs that comprise ionizable lipid, helper lipid, cholesterol, and poly(ethylene glycol) (PEG) lipid are currently the leading mRNA delivery systems with reliable safety and clinical transformation potential (*19*, *30*). Therefore, we designed and synthesized a novel ionizable lipid IC8 to prepare LNPs to encapsulate and deliver mRNA (Fig. 2A).

In general, LNPs can ensure adequate protein expression of mRNA in APCs. Furthermore, MHC I/MHC II complex presentation of relevant antigen by APCs and subsequent T cell priming are more critical for mRNA vaccines (*11*, *33*). However, the antigen presentation process of the MHC I complex is usually inefficient. Only 1 in 10,000 antigenic peptides is presented even for high-affinity MHC I ligands. Efficient antigen presentation of DCs requires sufficient protein expression and maturation (*12*–*14*). It has been reported that adjuvants can promote maturation of DCs and up-regulate the expression of costimulating molecules on the DCs such as CD80 and CD86, which can provide a secondary signal for activation of naive T cells (*35*, *47*). In particular, I-IFNs secreted from TLRs, retinoic acid–inducible gene-I–like receptors (RLRs), and STING pathway can promote the maturation of DCs and enhance immune responses (*21*, *48*–*50*). Compared to TLR or RLR agonists, STING agonists have superior safety, inducing relatively low levels of local and systemic inflammation. Several STING agonists have been developed for enhancing the immune response, such as Cyclic-di-GMP, ADU-S100 ammonium salt, dimethylxanthenylacetic acid, and Mn (*51*–*54*). Mn is a critical element in many physiological processes, which was found to activate the STING pathway by Jiang’s group in 2018 (*26*, *27*). However, it remains unclear whether Mn can enhance the immune effect of mRNA vaccines. Therefore, we introduced the adjuvant Mn and studied the function of Mn in mRNA vaccines (Fig. 1).

Although I-IFNs can enhance immune responses, excessive I-IFNs can inhibit mRNA translation (*25*, *44*). Therefore, it is crucial to balance the Mn content in mRNA vaccines. We first investigated the effect of Mn on mRNA expression in vitro and in vivo. The result showed that IC8/Mn LNPs containing different Mn contents could improve mRNA expression by promoting lysosomal escape compared to IC8 LNPs (Fig. 3, A to E). When the Mn content increased in IC8/Mn LNPs, the maturation of treated APCs improved but the transfection efficiency decreased owing to more IFN-β secretion by activating the STING pathway (Fig. 5, B and D to F). Therefore, IC8/Mn LNPs containing an appropriate dosage of Mn (Mn/mRNA 1:1, w/w) treated APCs achieved optimal antigen presentation owing to adequate protein expression and maturation of APCs (Fig. 5C). We used IC8/Mn LNPs loaded with Delta or Omicron mRNA to combat the SARS-CoV-2 variants. The results also demonstrated that IC8/Mn@D or IC8/Mn@O produced high and durable receptor binding domain (RBD)-specific antibodies in vaccinated mice, and IC8/Mn@D could efficiently neutralize pseudovirus of SARS-CoV-2 Delta variant (Fig. 4, A to E). Meanwhile, IC8/Mn@D [Mn/mRNA 1:1, w/w)induced more robust T_H_1-biased immune responses than IC8@D by stimulating more T_H_1-type cytokines in CD4^+^ T cells and CD8^+^ T cells extracted from the spleen and lymph nodes (Fig. 6, A to E).

In conclusion, we introduced a new adjuvant Mn into LNPs to construct a universal mRNA delivery system (IC8/Mn LNPs) that can both improve mRNA expression and activate the immune response. Especially grounded on simple adjuvant addition, IC8/Mn LNPs had good prospects for clinical translation.

## MATERIALS AND METHODS

### Materials

1,4-Bis(3-aminopropyl)piperazine and 1,2-epoxytetradecane were purchased from Heowns (Tianjin, China). MnCl_2_ was bought from Damao Chemical Reagent Factory (Tianjin, China). 1,2-Distearoyl-*sn*-glycero-3-phosphoethanolamine (DOPE), cholesterol (Chol), and 1,2-dimyristoyl-rac-glycero-3-methoxypolyethyleneglycol-2000 (DMG-PEG_2k_) were obtained from Avanti Polar Lipids Inc. (Alabaster, AL). 2-Mercaptoethanol, CPZ, and NS were purchased from Sigma-Aldrich (Shanghai, China). Granulocyte-macrophage colony-stimulating factor (GM-CSF) was obtained from PeproTech Inc. (New Jersey, USA). Commercial transfection reagent Lipofectamine 2000 (Lipo 2k), LysoTracker DND-26, and 4′,6-diamidino-2-phenylindole (DAPI) were obtained from Invitrogen (Massachusetts, USA). CD was purchased from Meilun Bio (Dalian, China). d-Luciferin potassium salt was purchased from Yeasen (Shanghai, China). Radioimmunoprecipitation assay (RIPA) lysis buffer and 3,3′,5,5′-tetramethylbenzidine (TMB) were purchased from Solarbio (Beijing, China). Protease inhibitor cocktails and SDS–polyacrylamide gel electrophoresis (SDS-PAGE) gels were purchased from Beyotime (Shanghai, China). The polyvinylidene difluoride membranes were purchased from Millipore (Massachusetts, USA). Glyceraldehyde-3-phosphate dehydrogenase (GAPDH; catalog no. 5174T), phospho-TBK1 (catalog no. 5483S), phospho–IRF-3 (catalog no. 4947S), and horseradish peroxidase (HRP)–linked anti-mouse IgG (catalog no. 7076S) were purchased from Cell Signaling Technology Inc. (Boston, USA). The p-TBK1 ELISA kit and p-IRF-3 ELISA kit were purchased from Jianglai Biotechnology Co. Ltd. (Shanghai, China). The SARS-CoV-2 Spike protein (319 to 541 amino acids) monoclonal antibody (catalog no. 67758-1-Ig) was purchased from Proteintech Group Inc. (Rosemont, USA). The goat anti-rabbit IgG-HRP (catalog no. abs20040) was purchased from Absin (Shanghai, China). The SARS-CoV-2 (B.1.617.2) Spike protein (S) RBD (His-Tag) (catalog no. CG220-01), and Bio-Lite Luciferase Assay Buffer (catalog no. DD1201-03-AA) were purchased from Vazyme Biotech Co. Ltd. (Nanjing, China). The SARS-CoV-2 Spike RBD (His-Tag) (B.1.1.529/Omicron)was purchased from ACROBiosystems Biotech Co. Ltd. (Beijing, China). The SARS-CoV-2 Spike (B.1.617.2) pseudotyped virus (GFP-luciferase) was purchased from Genomeditech Co. Ltd. (Shanghai, China). The CD16/32, phycoerythrin (PE) anti-mouse CD11c, fluorescein isothiocyanate (FITC) anti-mouse CD80, Allophycocyanin/Cyanine7 (APC-Cy7) anti-mouse CD86, APC anti-mouse SIINFEKL/H-2K^b^ 25-D1.16, PE anti-mouse CD4, Peridinin-Chlorophyll-Protein Complex (Percp) anti-mouse CD8α, FITC anti-mouse IFN-γ, PE/Cyanine7 (PE/Cy7) anti-mouse IL-2, APC anti-mouse IL-4, fixation buffer, and intracellular staining permeabilization wash buffer were purchased from BioLegend (California, USA). Dulbecco’s minimum essential medium (DMEM) and RPMI 1640 medium, fetal bovine serum (FBS), and PBS were purchased from Gibco (New York, USA). Our laboratory constructed and synthesized GFP mRNA, Luc mRNA, OVA mRNA, Delta mRNA, or Omicron mRNA. The Cy5 mRNA was purchased from TriLink BioTechnologies (California, USA). All other reagents and chemicals (analytical grade) were acquired from Damao Chemical Reagent Factory (Tianjin, China). DC2.4 cells acquired from the American Type Culture Collection were cultured in RPMI 1640 supplemented with 10% FBS and 1% penicillin-streptomycin at 37°C with 5% CO_2_. Human angiotensin-converting enzyme 2 (ACE2)-overexpressing cells (HEK293T-hACE2) acquired from Vazyme Biotech Co. Ltd. (Nanjing, China) were cultured in DMEM containing 10% FBS and 1% penicillin-streptomycin at 37°C with 5% CO_2_.

### Ethical statement for animal experiments

The animal experiments in this study have been approved by the Institutional Animal Care and Use Committee of West China Hospital, Sichuan University.

### Ionizable lipid synthesis

Crude IC8 was synthesized via the addition reaction of 1,4-bis(3-aminopropyl)piperazine and 1,2-epoxytetradecane in isopropyl alcohol at 90°C. Thirty-six hours later, the solvent was removed by rotary evaporation, and crude IC8 was purified by silica gel column chromatography with elution of dichloromethane:methanol = 20:1 (v/v) (containing 1% NH_4_OH) to obtain IC8. The construction of IC8 was confirmed using ^1^H nuclear magnetic resonance (fig. S1).

### In vitro transcription of mRNA

The mRNA was transcribed in vitro using a T7 High Yield RNA Transcription kit (Vazyme Biotech Co. Ltd., China) on a linearized DNA template with extra addition of pseudouridine and CleanCap AG (3′ OMe) (TriLink). The mRNA encoded the S protein of the SARS-CoV-2 Delta variant (B.1.617.2) or Omicron variant (B.1.1.529) with a 100-nucleotide-long poly (A) tail.

### Preparation and characterization of IC8@Luc and IC8/Mn@Luc

IC8@Luc and IC8/Mn@Luc were prepared using a microfluidic device (INano L, Micro&Nano Biologics Co. Ltd., China) with the mass ratio of IC8 to luciferase mRNA (Luc mRNA) set to 15:1. First, the ionizable lipid IC8, DOPE, Chol, and DMG-PEG_2k_ were dissolved in ethanol at a molar ratio of 35:16:46.5:2.5 as the organic phase. As an aqueous phase, different MnCl_2_ and mRNA mass ratios (0, 2.5, 5, and 10) were dissolved in 50 mM citrate buffer. Subsequently, IC8@Luc and IC8/Mn@Luc were obtained by mixing the organic phase and the aqueous phase [1:3 (v/v)] in the microfluidic device at a flow rate of 9 ml/min. Last, IC8@Luc and IC8/Mn@Luc were ultrafiltrated by a cup against 10 mM citrate buffer. After ultrafiltration, the content of Mn in IC8/Mn@Luc was approximately 20%, confirmed by Inductively Coupled Plasma-Atomic Emission Spectrometry (ICP-AES) (5100 synchronous vertical bidirectional observation (SVDV), Agilent, USA). Therefore, the final mass ratios of MnCl_2_ and mRNA in IC8/Mn@Luc were determined to be 0, 0.5, 1, and 2. The average particle size and zeta potential of IC8@Luc and IC8/Mn@Luc were determined using a Zetasizer Nano ZS90 (Malvern Instruments, Malvern, UK). The encapsulation efficiency of IC8@Luc and IC8/Mn@Luc was calculated by the following formula

$$\text{The \textbackslash{};encapsulation\textbackslash{}; efficiency}\;(\%)=\frac{\text{Total \textbackslash{};mRNA}-\text{Free\textbackslash{}; mRNA}}{\text{Total \textbackslash{};mRNA}}\times 100$$

wherein the total mRNA was determined by Stunner (Unchained Labs) (*55*). This method can skip the disruption step like Triton and quantify mRNA concentration according to ultraviolet/visible spectrometry owing to RNA absorbance most at 260 nm based on the Lambert-Beer law. Specifically, 3 μl of IC8@Luc and IC8/Mn@Luc was first added to the Stunner plate, respectively. Then, RNA-LNP under Gene Therapy was selected, and buffer was set as water. After detection, the concentration of mRNA in LNPs can be obtained. Moreover, the free mRNA was detected by the RiboGreen (Quant-iT RiboGreen RNA assay kit, Invitrogen). The morphologies of IC8@Luc and IC8/Mn@Luc with 2% phosphotungstic acid solution negative staining were observed by TEM (H-600, Hitachi, Japan).

### Extraction of BMDCs

BMDCs were extracted by the following steps. First, the tibia and femur of the hind legs were dissected from a euthanized C57BL/6 mouse (6 to 8 weeks). Then, bone marrow cells were obtained by rinsing the tibia and femur with the RPMI 1640 medium. Next, the cells were treated with precooled erythrocyte lysis solution for 5 min to remove red blood cells. After centrifugation (1200 rpm, 5 min), the cells were collected and cultured in a medium (containing 1% penicillin-streptomycin, 0.1% 2-mercaptoethanol, 10% FBS, 89% RPMI 1640 medium, and 1-μg GM-CSF of 50-ml medium).

### In vitro transfection of IC8@GFP and IC8/Mn@GFP

DC2.4 cells and BMDCs were seeded in 24-well plates (1 × 10^4^ cells per well for DC2.4 cells and 5 × 10^4^ cells per well for BMDCs) and cultured for 24 hours first. Then, DC2.4 cells and BMDCs were treated with IC8@GFP, IC8/Mn@GFP containing 0.5 μg of GFP mRNA, and IC8/Mn@Luc containing 0.5 μg of Luc mRNA (a scrambled mRNA control). The same dose of mRNA was complexed with the commercial transfection reagent Lipo 2k as a positive control. After incubation for 24 hours, inverted fluorescence microscopy (Nikon, Japan) and flow cytometry (NovoCyte, Eisen Bioscience, USA) were used to evaluate the transfection efficiency. All experiments were performed in triplicate.

### Cellular uptake

The cellular uptake experiment was performed to investigate whether the introduction of Mn into LNPs affected cellular internalization. DC2.4 cells were seeded in 24-well plates (1 × 10^4^ cells per well) overnight. The cells were treated with IC8@Cy5 and IC8/Mn@Cy5 (Mn/mRNA 1:1, w/w) encapsulating 0.5 μg of Cy5 mRNA. One, 2, 4, 6, 8, and 12 hours later, the cells were collected and rinsed twice with PBS for flow cytometry (NovoCyte, Eisen Bioscience, USA). All samples were carried out in triplicate.

### Mechanisms of cellular internalization

To further study whether adding Mn in IC8/Mn LNPs changed the cellular internalization pathway, DC2.4 cells were seeded in 24-well plates (1 × 10^4^ cells per well) overnight. Endocytosis inhibitors, including CPZ (10 μg/ml), CD (2.5 μg/ml), and NS (10 μg/ml), were added to cells before adding IC8@Cy5 and IC8/Mn@Cy5. Thirty minutes later, the cells were treated with IC8@Cy5 and IC8/Mn@Cy5 encapsulating 0.5 μg of Cy5 mRNA for an additional 2 hours. After that, the cells were collected and rinsed twice with PBS for flow cytometry (NovoCyte, Eisen Bioscience, USA). All samples were carried out in triplicate.

### Lysosomal escape

DC2.4 cells were inoculated in confocal dishes (1 × 10^5^ cells) overnight to study the lysosomal escape abilities of IC8 LNPs and IC8/Mn LNPs. IC8@Cy5, IC8/Mn@Cy5 containing 0.5 μg of Cy5 mRNA (red), and IC8/Mn@Luc (a scrambled mRNA control) were added to confocal dishes. After 3 hours of incubation, LysoTracker DND-26 (green, 75 μM) was added to confocal dishes to mark the lysosomes of cells. At 5 hours, the medium in the confocal dishes was discarded, and the cells were washed three times with precooled PBS to remove the LNPs in the medium and redundant lysosomal dyes. Subsequently, a 200-μl fixation buffer was used to fix the cells for 20 min. After washing the cells three times with precooled PBS, 1× intracellular staining permeabilization wash buffer containing 1% DAPI (blue) was applied to mark the nuclei of the cells. Ten minutes later, the cells were washed three times with precooled PBS again, and the lysosomal escape of IC8 LNPs and IC8/Mn LNPs was observed via confocal laser microscopy.

### Bioluminescence imaging

The in vivo distribution and expression of IC8 LNPs and IC8/Mn LNPs were evaluated using an In Vivo Imaging System (IVIS) Lumina system (PerkinElmer). IC8@Luc and IC8/Mn@Luc containing 20 μg of Luc mRNA were injected intramuscularly into male BALB/c mice (6 to 8 weeks old). At 6 hours after injection, d-luciferin potassium salt (150 mg/kg) was intraperitoneally injected into mice. Ten minutes later, the mice were euthanized, and bioluminescent imaging of the excised organs was performed using the IVIS Lumina system.

### Western blot

We verified the expression of Delta mRNA in IC8 LNPs and IC8/Mn LNPs. HEK293T cells were treated with IC8@D and IC8/Mn@D, respectively. After 24 hours of incubation, the cells were collected and then lysed in RIPA lysis buffer containing protease inhibitor cocktail to obtain TPs. Subsequently, proteins were separated by 8 to 15% SDS-PAGE gels. Later, the separated proteins were transferred to polyvinylidene difluoride membranes and then blocked for 2 hours with 5% skim milk. Then, the membranes were incubated with primary antibodies against GAPDH (1:1000) and SARS-CoV-2 S protein (319 to 541 amino acids) monoclonal antibody (1:5000) overnight at 4°C. After washing three times with TBST, the membranes were incubated with HRP-conjugated anti-rabbit secondary antibodies (goat anti-rabbit IgG-HRP; 1:5000 dilution) at room temperature for 2 hours. The Pierce ECL Western Blotting substrate was used to observe images by a ChemiScope 6200 Touch system (CLINX, China).

STING pathway activation of IC8/Mn LNPs was also determined by WB. The proteins were extracted from DC2.4 cells by referring to the above methods. The primary antibodies were GAPDH (1:1000), p-TBK1 (1:1000), and p-IRF-3 (1:1000) (two downstream proteins of the STING pathway). All other steps were the same as previously described.

### Immunization and sampling

BALB/c mice (6 to 8 weeks old) were casually divided into 11 groups (*n* = 6 per group). Mice received two intramuscular immunizations with a high (30 μg) or low (10 μg) dose of IC8@D, IC8/Mn@D, and SM102@D on day 0 and day 14. Mice in the placebo-immunized group were injected with PBS. Serum samples were collected on day 14, day 28, day 42, and day 56, inactivated at 60°C for 30 min, and then analyzed by ELISA. For IC8/Mn@O evaluation, the mice were injected intramuscularly twice with the high dose (30 μg of Omicron mRNA) on day 0 and day 14. The serum samples were collected at day 28, and the other experimental process was the same.

### Enzyme-linked immunosorbent assay

ELISA plates (high binding polystyrene, Corning) were coated with SARS-CoV-2 (B.1.617.2) Spike (S) protein RBD (His-Tag) or SARS-CoV-2 (B.1.1.529) Spike (S) protein RBD (His-Tag) at a final concentration of 1 μg/ml. After incubation overnight at 4°C, ELISA plates were washed four times with washing buffer and then blocked with 2% bovine serum albumin for 4 hours at 25°C. After washing twice with washing buffer, twofold serial dilutions of the serum samples were added and incubated overnight at 4°C. After washing four times with washing buffer, HPR-conjugated anti-mouse IgG (1:50,000 dilution) was added to the plates and incubated for 2 hours at 25°C. After another four washes with washing buffer, TMB was added to the plates. Thirty minutes later, the reactions were stopped with 2 M sulfuric acid. The absorbance was measured at 450 nm by a microplate reader (PerkinElmer, USA).

STING pathway activation of IC8/Mn LNPs was also determined by ELISA. The proteins were extracted from DC2.4 cells by referring to WB. The expressions of p-TBK1 and p-IRF-3 were detected by an ELISA kit.

### Pseudovirus neutralization assay

Neutralization assays against SARS-CoV-2 Spike (B.1.617.2) pseudotyped virus (GFP-luciferase) were performed to determine the pseudovirus neutralization ability of IC8@D and IC8/Mn@D. Briefly, the pseudovirus was diluted with DMEM (containing 10% FBS and 1% penicillin-streptomycin) at a total of 0.1 µl of pseudovirus. They were added to serial twofold diluted serum samples with DMEM (containing 10% FBS and 1% penicillin-streptomycin). After incubation for 1 hour at 37°C, HEK293T-hACE2 cells (3 × 10^4^ cells) were added to each well. The enzyme substrate was added for detecting luciferase activity by a microplate reader after incubation for 48 hours. The neutralization endpoint NT_50_ was defined as the fold dilution of serum necessary for 50% inhibition of luciferase activity compared with virus control samples.

### BMDC maturation and antigen presentation in vitro

OVA mRNA (mRNA encoding model antigen) was used to determine the maturation and antigen presentation ability of IC8 LNPs and IC8/Mn LNPs in vitro. BMDCs were cultured in 24-well plates (5 × 10^4^ cells per well) overnight. IC8@OVA and IC8/Mn@OVA containing 0.5 μg of OVA mRNA were added to each well and then incubated for 24 hours. Then, BMDCs were harvested and stained with PE anti-mouse CD11c, FITC anti-mouse CD80, APC/Cyanine7 anti-mouse CD86, and APC anti-mouse SIINFEKL/H-2K^b^ 25-D1.16 (a monoclonal antibody that specifically reacts with ovalbumin-derived peptide SIINFEKL bound to H-2K^b^ of MHC class I). After washing twice with PBS, flow cytometry (NovoCyte, Eisen Bioscience, USA) was used to detect the maturation and antigen presentation of BMDCs.

### DC maturation in the spleen and lymph nodes in vivo

To verify that IC8 LNPs and IC8/Mn LNPs could stimulate DCs maturation in the spleen and lymph nodes, BALB/c mice were euthanized after intramuscular injection of IC8@D and IC8/Mn@D (containing 30 μg of Delta mRNA) for 36 hours. Splenocytes and lymph node cells of mice were harvested and stained with PE anti-mouse CD11c, FITC anti-mouse CD80, and APC/Cyanine7 anti-mouse CD86. The maturation of DCs in the spleen and lymph nodes was executed by flow cytometry (NovoCyte, Eisen Bioscience, USA).

### ELISpot assay

We carried out an IFN-γ–based ELISpot assay with a Mouse IFN-γ ELISPOT^PLUS^ kit (ELISpot kit, Mabtech) to determine antigen-specific T cell responses. The relevant operations were as follows by referring to instructions. After washing four times with sterile PBS (200 μl per well), the plates were blocked with DMEM containing 10% FBS (200 μl per well) for at least 30 min at room temperature. Freshly extracted splenocytes (5 × 10^5^ cells per well) of IC8@D- and IC8/Mn@D-vaccinated mice at day 56 after immunization and the S protein peptide pools of the SARS-CoV-2 Delta variant (2 μg/ml of individual peptide) were added to the plates simultaneously. Unstimulated cells were used as negative control. The plates were incubated at 37°C and 5% CO_2_ for 36 hours. Subsequently, the cells were removed, and the plates were washed five times with PBS (200 μl per well). Later, the biotinylated IFN-γ detection antibody, the streptavidin-alkaline phosphatase (ALP) conjugate, and the substrate were added to the plates step by step, following the instructions. When the spots distinctly emerged, the development was stopped by rinsing the plates with deionized water. The numbers of the spots were obtained using an automatic ELISpot reader.

### ICS assay

Antigen-specific CD4^+^ T and CD8^+^ T immune responses were further determined by ICS assay. In summary, freshly extracted splenocytes and lymph node cells obtained from mice vaccinated with IC8@D and IC8/Mn@D on day 56 after immunization and S protein peptide pools of the SARS-CoV-2 Delta variant (2 μg/ml of individual peptide) were added to 24-well plates (2 × 10^6^ cells per well) simultaneously. After 2 hours, the monensin was added to each well to inhibit extracellular cytokine secretion. The cells were harvested 12 hours later and stained with PE anti-mouse CD4 and Percp anti-mouse CD8α for 40 min. Later, the cells were fixed with fixation buffer for 20 min. Last, the cells were permeabilized in 1× permeabilizing buffer and stained with FITC anti-mouse IFN-γ, PE/Cyanine7 anti-mouse IL-2, and APC anti-mouse IL-4. After being washed twice with PBS, the cells were analyzed by flow cytometry (NovoCyte, Eisen Bioscience, USA).

### Safety and Stability profiles of IC8/Mn@D

An acute toxicity test was used to evaluate the preliminary safety of IC8/Mn@D in comparison with IC8@D. Male BALB/c mice were treated with IC8@D and IC8/Mn@D containing 30 μg of Delta mRNA by intramuscular administration. Twenty-four hours later, the serum samples were obtained through centrifugation. Related biochemical indicators including ALT, AST, TP, ALB, CRE, CK, TRIGL, and UREA were detected by an automatic hematological biochemical analyzer (Hitachi High-Technologies Corp., Minato-ku, Tokyo, Japan). The organs of each mouse were harvested from IC8@D- and IC8/Mn@D-immunized mice at day 56 in the previous experiment for H&E staining. All slide images were taken with an Olympus-BX 43 fluorescence microscope (Olympus Corp., Tokyo, Japan).

IC8/Mn@D (Mn/mRNA 1:1, w/w) was stored at 4°C for 0, 7, 14, and 28 days to study its stability by observing the change of the particle size, zeta potential, mRNA encapsulation efficiency, and IgG titer. Mice (*n* = 6) were injected IC8/Mn@D (Mn/mRNA 1:1, w/w) intramuscularly twice with a high dose (30 μg Delta mRNA) on day 0 and day 14. The serum samples were collected at day 28 and analyzed by ELISA.

### Statistical analysis

Statistical analyses were performed using GraphPad Prism 8.01 software. The one-way analysis of variance (ANOVA) was used to assess the significance of differences among groups. The significance was defined as follows: *P < 0.05, **P < 0.01, ***P < 0.001, and ****P < 0.0001.

## Notes

View/request a protocol for this paper from *Bio-protocol*.
