# Supplementary material for: Manganese-coordinated mRNA vaccines with enhanced mRNA expression and immunogenicity induce robust immune responses against SARS-CoV-2 variants
Source: Sci Adv. 2022 Dec 23;8(51):eabq3500. doi: 10.1126/sciadv.abq3500 (PMC9788765; doi:10.1126/sciadv.abq3500)
Supplement: Supplementary file 1 — Figs. S1 to S8 Table S1 [file sciadv.abq3500_sm.pdf]

Science Advances
AAAS

Supplementary Materials for

Manganese-coordinated mRNA vaccines with enhanced mRNA expression and immunogenicity induce robust immune responses against SARS-CoV-2 variants

Na Fan *et al.*

Corresponding author: Xiangrong Song, songxr@scu.edu.cn

Sci. Adv. **8**, eabq3500 (2022)
DOI: 10.1126/sciadv.abq3500

This PDF file includes:

Figs. S1 to S8
Table S1

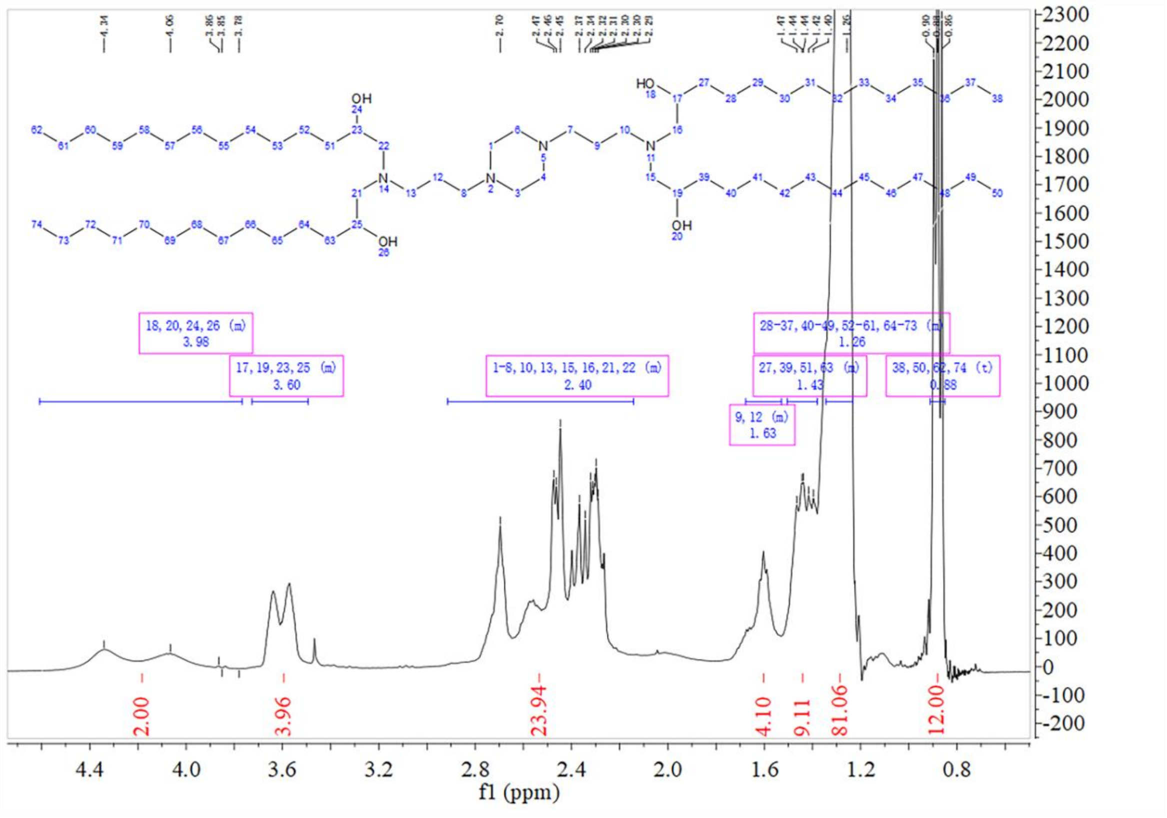

Fig. S1. ^{1}H NMR spectrum of of IC8.

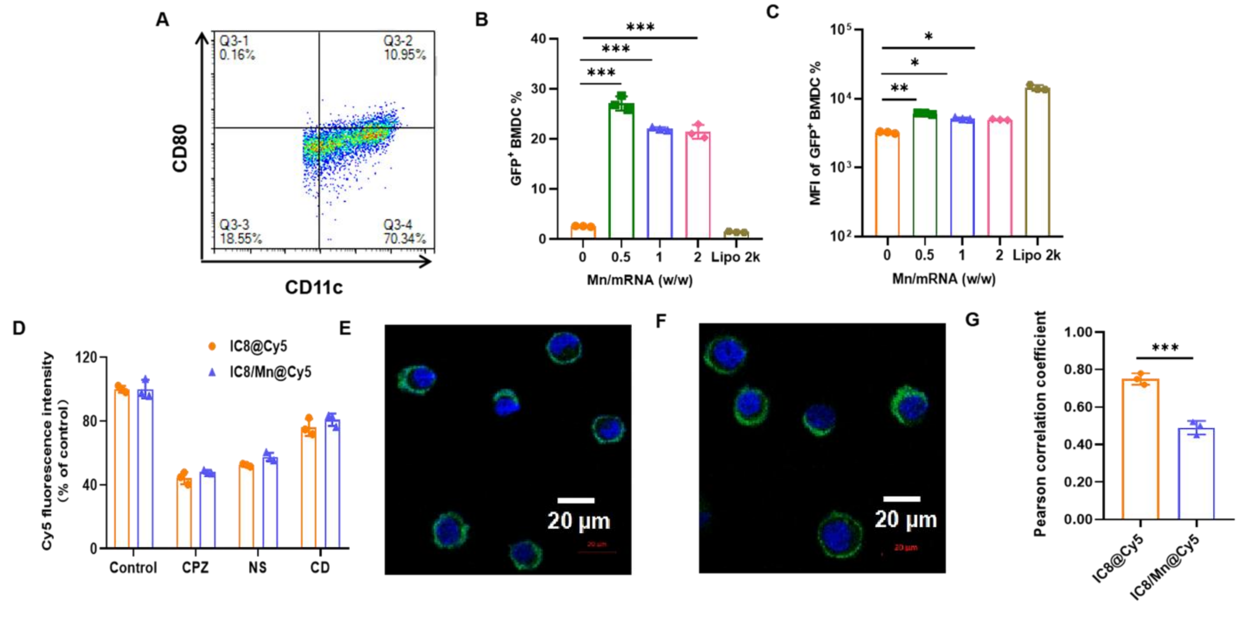

Fig. S2. *In vitro* and *in vivo* delivery of IC8/Mn@mRNA. A) The purity and maturity of BMDCs at day 5. B-C) *In vitro* transfection results of IC8@GFP and IC8/Mn@GFP in BMDCs by flow cytometry. D) Cellular uptake pathway of IC8@Cy5 and IC8/Mn@Cy5. E) The confocal

fluorescence image of DC2.4 cells incubated with IC8/Mn@Luc in 10% FBS medium for 5 h. DAPI (blue) was used to track the cell nuclei, and LysoTracker Green DND-26 (green) was used to track lysosomes. F) The confocal fluorescence image of DC2.4 cells incubated with IC8/Mn@Cy5 in 10% FBS medium for 0 h. DAPI (blue) was used to track the cell nuclei, and LysoTracker Green DND-26 (green) was used to track lysosomes. G) The pearson's correlation coefficient of green and red signals in Fig. 3C. The data are shown as the mean ± SD. Statistical significance was tested using ANOVA among groups. $^{*}P < 0.05$, $^{**}P < 0.01$, $^{***}P < 0.001$.

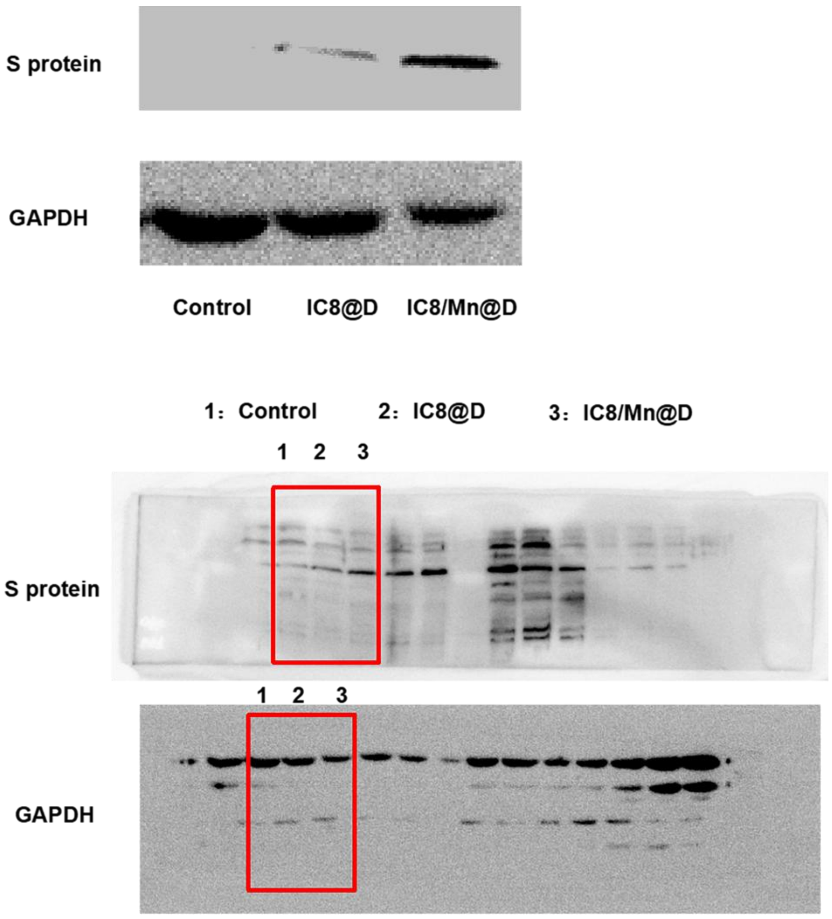

Fig. S3. Expression of IC8/Mn@D in 293T cells.

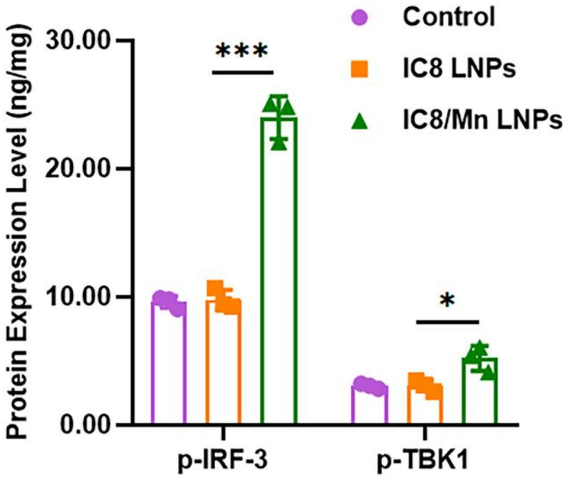

Fig. S4. ELISA analysis of the STING pathway downstream protein expressions (p-IRF-3 and p-TBK1) in DC 2.4 cells after treatment with PBS, IC8 LNPs and IC8/Mn LNPs for 24 h.

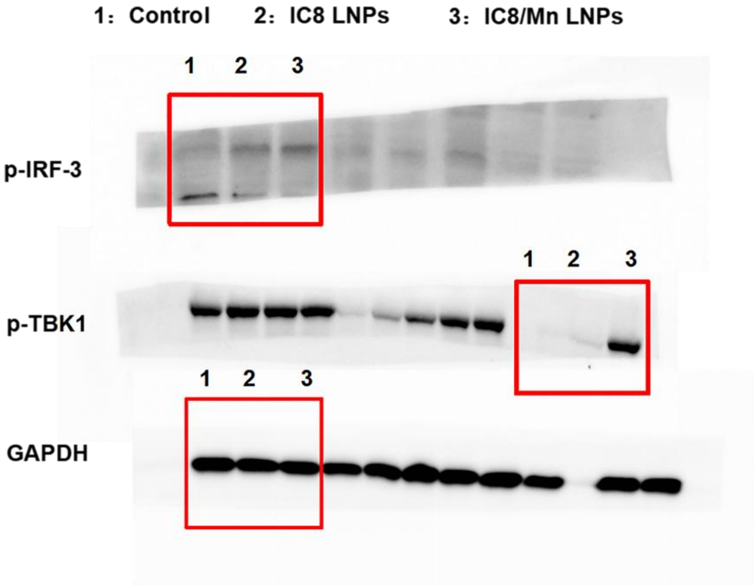

Fig. S5. Uncropped western blot images of the STING pathway downstream protein expressions (p-IRF-3 and p-TBK1) in DC 2.4 cells after treatment with PBS, IC8 LNPs and IC8/Mn LNPs for 24 h.

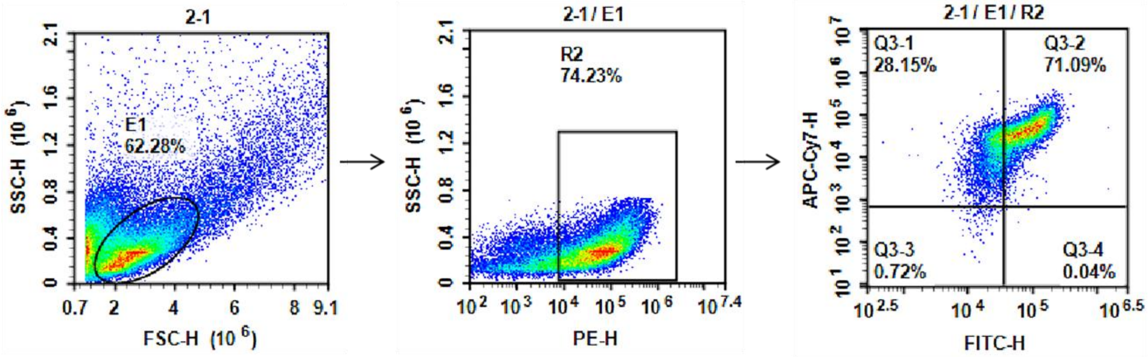

Fig. S6. Representative gating strategy of DCs maturation (CD11c$^+$CD80$^+$CD86$^+$) *in vitro* and *in vivo*.

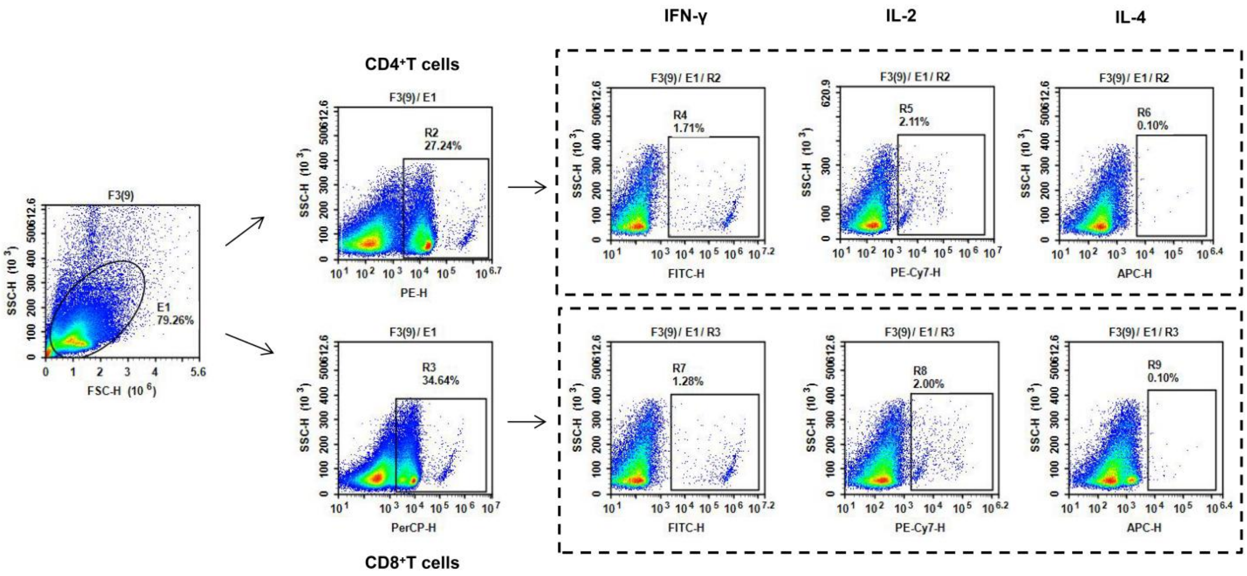

Fig. S7. Representative gating strategy of intracellular cytokine staining in splenocytes and lymph node cells.

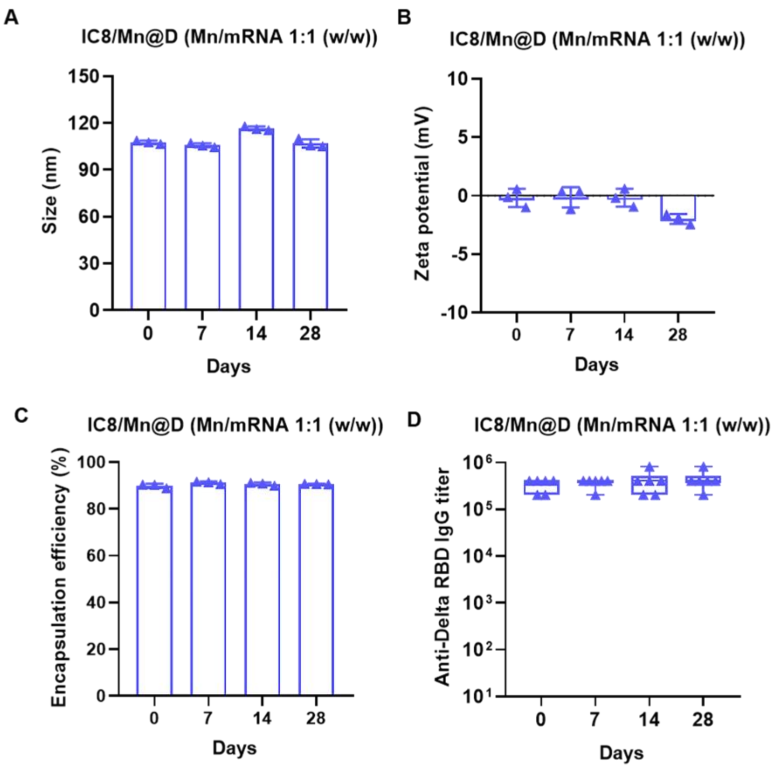

Fig. S8. Particle size (A), zeta potential (B), mRNA encapsulation efficiency (C) and IgG titer (D) (BALB/c mice were immunized twice and serum samples were collected at day 28) were detected after IC8/Mn@D (Mn/mRNA 1:1 (w/w)) storage at 4°C for 0, 7, 14 and 28 days.

Table S1. The formulations of IC8@mRNA and IC8/Mn@mRNA.

	IC8	DOPE	Chol	DMG-PEG_{2k}	IC8/mRNA (w/w)	**Mn/mRNA (w/w)**	30 μg mRNA Theoretical Mn (μg)	30 μg mRNA Measured Mn (μg)
IC8@mRNA	35	16	46.5	2.5	15	**0**	0	0
	35	16	46.5	2.5	15	**0.5**	75	14.2
IC8/Mn@mRNA	35	16	46.5	2.5	15	**1**	150	27
	35	16	46.5	2.5	15	**2**	300	62.3
